# Selenium status in UK pregnant women and its relationship with hypertensive conditions of pregnancy

**DOI:** 10.1017/S000711451400364X

**Published:** 2015-01-28

**Authors:** Margaret P. Rayman, Sarah C. Bath, Jacob Westaway, Peter Williams, Jinyuan Mao, Jessica J. Vanderlelie, Anthony V. Perkins, Christopher W. G. Redman

**Affiliations:** 1 Department of Nutritional Sciences, Faculty of Health and Medical Sciences, School of Biosciences and Medicine, University of Surrey, GuildfordGU2 7XH, UK; 2 School of Medical Science, Griffith Health Institute, Griffith University, Queensland, QLD4222, Australia; 3 Department of Mathematics, Faculty of Engineering and Physical Sciences, University of Surrey, GuildfordGU2 7XH, UK; 4 Nuffield Department of Obstetrics and Gynaecology, University of Oxford, OxfordOX3 9DU, UK

**Keywords:** Selenium status, Pregnancy, Hypertension, Hypertensive conditions of pregnancy

## Abstract

Dietary intake/status of the trace mineral Se may affect the risk of developing hypertensive conditions of pregnancy, i.e. pre-eclampsia and pregnancy-induced hypertension (PE/PIH). In the present study, we evaluated Se status in UK pregnant women to establish whether pre-pregnant Se status or Se supplementation affected the risk of developing PE/PIH. The samples originated from the SPRINT (Selenium in PRegnancy INTervention) study that randomised 230 UK primiparous women to treatment with Se (60 μg/d) or placebo from 12 weeks of gestation. Whole-blood Se concentration was measured at 12 and 35 weeks, toenail Se concentration at 16 weeks, plasma selenoprotein P (SEPP1) concentration at 35 weeks and plasma glutathione peroxidase (GPx3) activity at 12, 20 and 35 weeks. Demographic data were collected at baseline. Participants completed a FFQ. UK pregnant women had whole-blood Se concentration lower than the mid-range of other populations, toenail Se concentration considerably lower than US women, GPx3 activity considerably lower than US and Australian pregnant women, and low baseline SEPP1 concentration (median 3·00, range 0·90–5·80 mg/l). Maternal age, education and social class were positively associated with Se status. After adjustment, whole-blood Se concentration was higher in women consuming Brazil nuts (*P*= 0·040) and in those consuming more than two seafood portions per week (*P*= 0·054). A stepwise logistic regression model revealed that among the Se-related risk factors, only toenail Se (OR 0·38, 95 % CI 0·17, 0·87, *P*= 0·021) significantly affected the OR for PE/PIH. On excluding non-compliers with Se treatment, Se supplementation also significantly reduced the OR for PE/PIH (OR 0·30, 95 % CI 0·09, 1·00, *P*= 0·049). In conclusion, UK women have low Se status that increases their risk of developing PE/PIH. Therefore, UK women of childbearing age need to improve their Se status.

As many as 10 % of women are affected by high blood pressure in pregnancy and some 2–5 % will go on to develop proteinuria, triggering a diagnosis of the more serious hypertensive condition, i.e. pre-eclampsia (PE)^(^ ^1^ ^,^ ^2^ ^)^. Not only is PE associated with high maternal and fetal morbidity and mortality^(^ ^1^ ^,^ ^2^ ^)^, women who have had PE, or indeed pregnancy-induced hypertension (PIH), have a greater risk of developing hypertension, stroke and IHD in later life^(^ ^3^ ^–^ ^5^ ^)^. Furthermore, they have daughters who are at an increased risk of developing the same pregnancy complications^(^ ^6^ ^,^ ^7^ ^)^ and children who are more likely to develop hypertension as adults^(^ ^8^ ^)^.

There are indications that dietary intake or status of the trace mineral Se may affect the risk of developing hypertensive conditions of pregnancy^(^ ^9^ ^)^. For instance, Chinese women supplemented with Se have been shown to have a lower risk of developing PIH^(^ ^10^ ^)^. A negative correlation has been found between Se status and the incidence of PE in an epidemiological study of forty-five countries^(^ ^11^ ^)^. Significantly lower levels of selenoenzymes such as glutathione peroxidase (GPx) and thioredoxin reductase have been found in serum, plasma and placenta samples from pre-eclamptic women than in those from matched healthy controls^(^ ^12^ ^–^ ^15^ ^)^. Genetic evidence suggests that the anti-inflammatory selenoprotein S (SELS) affects the risk of developing PE^(^ ^16^ ^)^.

In a previous UK study, we have found that the concentration of Se in toenails (laid down from 3 to 12 months previously) of women with PE is significantly lower than that of matched controls (*P*= 0·001)^(^ ^17^ ^)^. That study triggered a pilot trial of Se supplementation in UK pregnant women that aimed to reduce biomarkers of PE risk^(^ ^9^ ^)^. In that trial, we showed a significantly lower (*P*= 0·039) concentration of plasma soluble vascular endothelial growth factor receptor-1 (sFlt-1), a recognised biomarker of pre-eclampsia risk, at 35 weeks in the Se-treated group (60 μg/d as Se yeast) than in the placebo group in participants of low Se status (lowest quartile) at baseline^(^ ^9^ ^)^.

The present study used samples and data collected in that pilot trial. To establish the Se status, we used a range of measures at various gestational ages: (1) whole-blood Se concentration at baseline (12 weeks) and after 23 weeks of treatment with Se or placebo (35 weeks); (2) toenail Se concentration in clippings collected at 16 weeks (a measure of pre-pregnancy Se status); (3) plasma glutathione peroxidase (GPx3) activity at 12, 20 and 35 weeks; (4) plasma selenoprotein P (SEPP1) concentration at 35 weeks.

Our two hypotheses were that (1) UK pregnant women have low Se status, as determined by a number of parameters, and (2) pre-pregnant Se status or Se supplementation in pregnancy affects the risk of developing hypertensive conditions of pregnancy, i.e. PE or PIH, as a single outcome (PE/PIH).

## Experimental methods

The selection of subjects has been described previously^(^ ^9^ ^)^. Women were excluded if they were under 18 years, current smokers, taking any supplement containing Se, taking thyroid medication, had a multi-fetal pregnancy or a number of other specified pregnancy complications, or withheld consent. Blood, plasma and toenail samples originated from the SPRINT (Selenium in PRegnancy INTervention) study (trial registration no. ISRCTN37927591) that randomised 230 primiparous women in Oxford, UK, to treatment with Se (60 μg/d Se, as Se yeast) or placebo (placebo yeast) from their first hospital antenatal visit (mean gestational age 12·3 weeks) until the delivery of the baby^(^ ^9^ ^)^. Blood samples, from which plasma was prepared, were collected at baseline (12 weeks), 20 and 35 weeks, while toenail clippings were collected at 16 weeks, as described previously^(^ ^9^ ^)^.

A FFQ was administered at recruitment and was completed by 219 women (95·6 % of the cohort). Meanwhile, clinical and demographic data, including weight and height (for calculation of BMI at baseline), date of birth (for calculation of maternal age at recruitment), age at which the education of the mother ceased, and occupation were recorded in order to explore the potential effect of a number of factors on Se status and on the development of PE/PIH.

PIH was defined as new hypertension appearing for the first time after 20 weeks of pregnancy. Hypertension was defined as diastolic blood pressure (DBP) ≥ 90 mmHg on two occasions (4 h apart). PE was defined as PIH and new-onset proteinuria after 20 weeks of pregnancy ( ≥ 300 mg/24 h or ≥ 2+ dipstick mid-stream urine/catheter specimen of urine). A protein:creatinine ratio of >30 mg/ mmol of creatinine was generally used to confirm proteinuria. These criteria are an extension of those adopted by the International Society for the Study of Hypertension in Pregnancy^(^ ^9^ ^,^ ^18^ ^)^.

The present study was conducted according to the guidelines laid down in the Declaration of Helsinki, and all procedures involving human subjects were approved by the Milton Keynes Research Ethics Committee (REC reference no. 08/H0603/46). Written informed consent was obtained from all subjects.

### Selenium status measurements

Whole-blood samples were stored at − 20°C until thawed for analysis, and were analysed in duplicate. Se concentration was measured with full quality-control procedures by inductively coupled plasma-MS as described previously^(^ ^9^ ^)^. The CV for the blood Se assay was 0·25 % at 1·4 μmol/l and 0·17 % at 3·0 μmol/l.

Toenail Se concentration was measured in clippings collected from all ten toes at 16 weeks. Toenails were prepared for analysis as described previously^(^ ^17^ ^)^. Se content was determined using instrumental neutron activation analysis conducted at the Interfaculty Reactor Institute in Delft as described previously^(^ ^17^ ^)^. The analysis of NIST SRM 1577b Bovine Liver gave a mean and combined standard uncertainty (forty-five determinations) of 0·73 (sd 0·005) mg/kg, compared with a certified mean of 0·74 (sd 0·02) mg/kg, indicating excellent accuracy of the method. The laboratory has an embedded quality-control system for quality assurance and management, which complies with the requirements of the International Standard ISO/IEC 17025:2005 and has been accredited by the Dutch Council for Accreditation since 1993.

SEPP1 concentrations were measured in the laboratory of Raymond Burk at the University of Vanderbilt using an ELISA as described previously^(^ ^19^ ^)^.

GPx3 activity was measured in plasma samples obtained at 12, 20 and 35 weeks at Griffith University as described previously^(^ ^13^ ^)^. Briefly, GPx activity was measured spectrophotometrically in triplicate in 10 μl plasma samples. The rate of NADPH oxidation was recorded at 340 nm over 5 min using a Tecan Sunrise Absorbance Reader with Magellan Standard software (Tecan). The activity of GPx was calculated as units/l of plasma (one unit of activity was defined as 1 μmol of NADPH oxidised per min).

### Predictors of selenium status

#### Demographic factors

Factors considered to be likely to affect Se status were as follows: baseline BMI; maternal age at recruitment; age at which the education of the mother ceased; occupation; ethnicity (Caucasian/other); smoking status (never smoked/ex-smoker). Occupation was used to code maternal social class according to the National Readership Demographic categories^(^ ^20^ ^)^ and was classified into two groups: (1) A and B (middle class and above) and (2) C1 to E (lower middle class and below). BMI was coded as < 25 or ≥ 25 kg/m^2^.

#### Dietary factors

The FFQ containing eighteen items was based on the EPIC-Norfolk FFQ^(^ ^21^ ^)^, and was designed to collect information on the consumption of the following Se- and iodine-rich foods: seafood (white, oily and shellfish, fish fingers, and fish roe); meat (beef, beef burgers, pork and lamb, bacon, ham, sausages, and corned beef); poultry; Brazil nuts; offal (liver and liver products); dairy products (grouped as one item in the questionnaire); milk. Where participants had not given a frequency of consumption for individual food items in the FFQ (*n* 7), these foods were coded as ‘never or rarely’. The answers were converted to weekly portions, and for seafood, meat and poultry, the portions were summed to give a total. Food items were then recoded to reflect a high and low intake of the food item, i.e. intake above and below the median. For liver products and Brazil nuts, participants were dichotomised into either ‘consumers’ (any frequency of consumption) or ‘non-consumers’ (those who answered as ‘never/rarely’ consuming the products) due to small number of consumers.

### Statistical analyses

Whole-blood Se, toenail Se and SEPP1 concentrations and GPx3 activity were not normally distributed, hence data are presented as medians and range values. Mann–Whitney *U* tests were used to compare the concentrations/activity between the Se-treated and placebo groups.

The Wilcoxon matched-pair test was used to compare the changes in GPx3 activity from 12 to 20 weeks, 12 to 35 weeks and 20 to 35 weeks, and the change in whole-blood Se concentration from 12 to 35 weeks in the Se-treated and placebo groups.

Correlations were analysed by Spearman's rank correlation test. The Mann–Whitney test was used to compare the differences in whole-blood Se and toenail Se concentrations between the groups. A general linear model was used on log-transformed whole-blood Se and toenail Se concentrations to adjust the dietary analysis by those demographic factors that were significantly associated with Se status in univariate analysis. A series of models was constructed where each individual dietary factor was entered separately into a model, with social class, maternal age and age at which the education of the mother ceased being confounders.

We explored the effect of known^(^ ^7^ ^)^ and potential Se-related risk factors individually on the development of PE and PIH combined (PE/PIH) by multiple logistic regression. Factors that showed significance individually were then entered into a forward logistic regression model.

#### Subgroup analysis by compliance

A small number of women (*n* 9) in the Se-treated group took very few tablets ( ≤ 23 %). All the other women took 60 % or more of those they could have taken (expressed as a percentage of the number of days between starting treatment and the delivery of the baby); hence, the forward logistic regression modelling was repeated after excluding Se-treated women who took < 60 % of their treatment pills.

Significance was set at *P*< 0·05, and analyses were conducted using the Statistical Package for the Social Sciences (version 21.0; SPSS, Inc.).

## Results

### Selenium status

#### Baseline measurements

Toenail Se concentration (16 weeks) and pre-treatment GPx3 activity (12 weeks) are presented in Table 1. There were no significant differences observed between the two treatment groups at baseline. Previously published^(^ ^9^ ^)^ pre-treatment values of whole-blood Se concentration (12 weeks) in the same study are also presented in Table 1 to complete the dataset of Se status.

**Table 1:** Selenium status in UK pregnant women at 12 (baseline), 20 and 35 weeks of gestation (Median values and ranges)

			Measurements	
			All participants	Placebo group	Se group	
Parameters	Women (*n*)	Week of gestation	Median	Range	Median	Range	Median	Range	*P* † (placebo group *v.* Se group)
Whole-blood Se concentration (μmol/l)‡	229	12	1·31	0·84–3·33	1·32	0·84–2·19	1·31	0·95–3·33	0·565
Toenail Se concentration (μg/g)§	218	16	0·61	0·46–1·11	0·60	0·46–1·10	0·62	0·46–1·11	0·387
GPx3 activity (units/l)	227	12	72·4	42·5–141·9	72·0	52·6–138·8	72·7	42·5–141·9	0·601
GPx3 activity (units/l)	221	20	NA	75·4	46·6–113·8	78·2*	43·9–169·7	0·225
GPx3 activity (units/l)	215	35	NA	73·4	47·3–126·8	75·4**	42·4–139·3	0·125
Whole-blood Se concentration (μmol/l)‡	215	35	NA	1·16***	0·69–2·15	1·87***	1·05–3·73	< 0·0001
Plasma SEPP1 concentration (mg/l)‡	215	35	NA	3·00	0·90–5·80	5·30	2·40–7·40	< 0·0001

GPx3, plasma glutathione peroxidase; NA, not applicable; SEPP1, selenoprotein P.

*P value for difference in GPx activity in Se group by Wilcoxon matched pairs test (12 *v.* 20 weeks) = 0.025; **P value for difference in GPx activity in Se group by Wilcoxon matched pairs test (12 *v.* 35 weeks) = 0.014; ***P value for difference in whole-blood Se in placebo and Se groups by Wilcoxon matched pairs test (12 *v.* 35 weeks) < 0.0001.

† *P* values were obtained from the Mann–Whitney *U* test comparing the differences between the treatment groups.

‡ Previously published data from the same study^(^ ^9^ ^)^. The *P* value for whole-blood Se concentration at 12 weeks differs from that reported in Rayman *et al.* ^(^ ^9^ ^)^ where data were log-transformed and a parametric test was used.

§ Toenails were clipped at approximately 16 weeks of gestation so Se concentration in clippings would have been unaffected by Se treatment and reflects pre-pregnancy status.

**Fig. 1:**
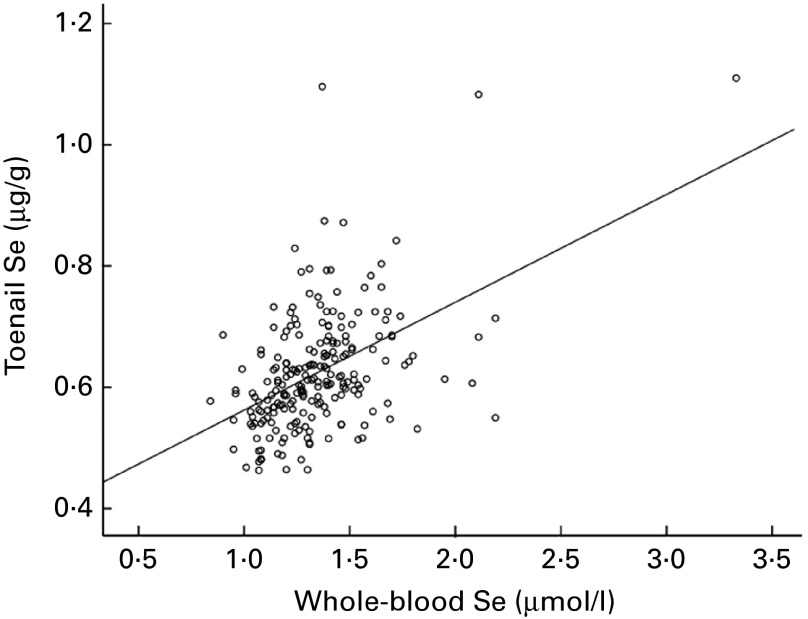
Correlation between whole-blood selenium concentration at 12 weeks and toenail selenium concentration at 16 weeks (Spearman's ρ = 0·450, *P*< 0·001) in UK pregnant women.

Baseline whole-blood Se concentration (12 weeks) was significantly correlated with the concentration of Se in toenails clipped at 16 weeks (Spearman's ρ = 0·447, *P*< 0·001), as shown in Fig. 1, but not with GPx3 activity measured at 12 weeks (Spearman's ρ = 0·042, *P*= 0·533).

Toenail Se concentration was significantly negatively, albeit weakly, correlated with DBP at baseline (Spearman's ρ = − 0·135, *P*= 0·047).

#### Effect of treatment on selenium status and plasma glutathione peroxidase activity

Table 1 presents the data for GPx3 activity measured at 20 and 35 weeks. Previously published^(^ ^9^ ^)^ Se concentration in whole blood at 35 weeks and SEPP1 concentration in plasma at 35 weeks following treatment with Se or placebo are also presented for completeness.

There was no significant difference observed in GPx3 activity between the Se-treated and placebo groups at either 20 or 35 weeks. However, using the Wilcoxon matched-pair test, GPx3 activity increased in the Se-treatment group only, from 12 to 20 weeks (*P*= 0·025) and from 12 to 35 weeks (*P*= 0·014), although there was no further increase in the activity from 20 to 35 weeks (*P*= 0·219).

#### Effect of demographic and dietary factors on selenium status

Baseline whole-blood Se and toenail Se concentrations were significantly correlated, though weakly, with maternal age (Spearman's ρ = 0·18, *P*= 0·006 and ρ = 0·16, *P*= 0·018, respectively) and with age at which the education of the mother ceased (Spearman's ρ = 0·17, *P*= 0·009 and ρ = 0·20, *P*= 0·004, respectively).

The effect of other demographic and dietary factors on Se status is summarised in Table 2. Women who were classified as middle class and above had a significantly higher Se status as measured in both whole blood and toenails than those classified as lower middle class and below. Se status was not significantly affected by smoking status (never smoker or ex-smoker), ethnicity or BMI.

**Table 2:** Predictors of selenium status (Number of women; median values and interquartile ranges)

	12-week whole-blood Se (μmol/l)	Toenail Se (μg/g)
	Women (*n*)	Median	Interquartile range	*P*	Adjusted *P* *	Women (*n*)	Median	Interquartile range	*P*	Adjusted *P* *
Demographic factors										
Social class										
Middle and above	101	1·33	1·22–1·48	0·027	NA†	98	0·63	0·59–0·69	0·004	NA†
Lower middle and below	128	1·30	1·14–1·45			120	0·60	0·55–0·65		
Smoking status										
Never smoked	156	1·32	1·19–1·47	0·529	NA†	148	0·61	0·57–0·67	0·261	NA†
Ex-smoker	73	1·30	1·18–1·46			70	0·61	0·54–0·66		
Ethnicity										
Caucasian	213	1·31	1·18–1·47	0·438	NA†	202	0·61	0·56–0·67	0·869	NA†
Other	16	1·34	1·25–1·48			16	0·62	0·55–0·63		
BMI										
< 25 kg/m^2^	138	1·35	1·19–1·48	0·183	NA†	131	0·61	0·56–0·67	0·841	NA†
≥ 25 kg/m^2^	91	1·29	1·16–1·44			87	0·61	0·56–0·68		
Dietary factors										
Seafood										
< 2 portions/week	98	1·27	1·14–1·41	0·010	0·054	95	0·59	0·54–0·65	0·012	0·078
≥ 2 portions/week	121	1·36	1·21–1·48			118	0·63	0·57–0·69		
Meat										
< 7 portions/week	108	1·33	1·19–1·47	0·540	0·299	107	0·61	0·56–0·66	0·729	0·610
≥ 7 portions/week	111	1·30	1·16–1·46			106	0·61	0·56–0·68		
Brazil nuts										
Non-consumer	193	1·30	1·16–1·46	0·005	0·040	187	0·60	0·56–0·66	0·034	0·147
Consumer	26	1·40	1·31–1·61			26	0·66	0·60–0·72		
Offal (liver)										
Non-consumer	182	1·31	1·18–1·47	0·583	0·765	176	0·61	0·56–0·66	0·702	0·804
Consumer	37	1·35	1·22–1·45			37	0·62	0·56–0·72		
Dairy products										
≤ 1 portion/d	110	1·30	1·16–1·45	0·423	0·672	107	0·61	0·56–0·67	0·916	0·112
>1 portion/d	109	1·32	1·19–1·48			106	0·61	0·57–0·67		
Cows' milk										
< 280 ml/d	146	1·32	1·18–1·46	0·920	0·983	144	0·60	0·56–0·66	0·016	0·083
≥ 280 ml/d	73	1·32	1·21–1·46			69	0·63	0·57–0·70		

NA, not applicable.

* Conducted on log-transformed Se variables; a series of general linear models were constructed where each individual dietary factor was entered into a separate model, with maternal age, age at which the education of the mother ceased and social class (dichotomised) being confounders.

† Demographic factors were used to adjust the *P* values for the dietary factors only.

Whole-blood and toenail Se concentrations were significantly higher in consumers of Brazil nuts (*P*= 0·005 and *P*= 0·03, respectively); however, after adjusting for demographic factors, the difference in toenail Se concentration lost significance. Women who reported consuming at least two portions of seafood (white fish, oily fish or other seafood) per week had a significantly higher concentration of Se in both whole blood and toenails (*P*= 0·001 for each); the significance disappeared after adjustment, but remained close to significance for whole-blood Se concentration (*P*= 0·054). Intake of dairy products, meat and poultry, or liver did not significantly affect Se status (whole blood and toenails). Although intake of cows' milk was associated with toenail Se status, this association lost significance after adjustment.

### Hypertensive conditions of pregnancy

Data on pregnancy outcome were available for 227 women, twenty of whom developed a hypertensive condition (PE *n* 11 and PIH *n* 9).

#### Factors affecting the development of pregnancy-induced hypertension/pre-eclampsia

We explored the effect of a number of relevant factors on the risk of developing PE/PIH. DBP, maternal age, history of PE in mother or sister, and BMI are known risk factors for the development of PE or PIH^(^ ^7^ ^)^. We also investigated the effect of parameters of Se status (whole-blood Se concentration at 12 and 35 weeks, toenail Se concentration at 16 weeks, and SEPP1 concentration at 35 weeks) and treatment (Se *v.* placebo). Logistic regression on risk factors assessed individually showed that DBP, BMI and toenail Se concentration were the only factors that significantly affected the combined PE/PIH outcome; both SEPP1 concentration and treatment failed to reach significance (see Table 3). Although social class was significantly associated with 12-week whole-blood Se and toenail Se concentrations (Table 2), it was not significantly associated with PE/PIH (*P*= 0·396) and was therefore excluded from the model. When a stepwise model was run with only significant or near-significant factors entered into the model (SEPP1 was excluded as it was highly associated with treatment: mean 5·13 *v*. 2·95 mg/l for the Se-treated and placebo groups, respectively, *P*< 0·0005), baseline DBP (OR 1·10, 95 % CI 1·03, 1·18), BMI (OR 1·13, 95 % CI 1·00, 1·27) and toenail Se concentration (OR 0·38, 95 % CI 0·17, 0·87) remained significant, with treatment again failing to reach significance (*P*= 0·067) (adjusted model 1; Table 3). However, when the model was rerun after the non-compliers were excluded (i.e. Se-treated women who took < 60 % of their treatment pills; for explanation see the Experimental methods section), treatment (Se *v*. placebo) became significant (OR 0·297, 95 % CI 0·089, 0·995, *P*= 0·049) (adjusted model 2; Table 3).

**Table 3:** Unadjusted and adjusted risk factors for the development of pre-eclampsia (PE)/pregnancy-induced hypertension (PIH) determined by logistic regression (Odds ratios and 95 % confidence intervals).

	Unadjusted model*	Adjusted model 1†	Adjusted model 2‡
Risk factors	Women (*n*)	OR	95 % CI	*P*	Women (*n*)	Adjusted OR	95 % CI	*P*	Women (*n*)	Adjusted OR	95 % CI	*P*
Baseline diastolic BP	227	1·14	1·07, 1·21	< 0·0005	217	1·10	1·03, 1·18	0·005	210	1·12	1·04, 1·20	0·002
BMI (kg/m^2^)	227	1·20	1·09, 1·32	< 0·0005	217	1·13	1·00, 1·27	0·049	210	1·08	0·96, 1·22	0·214
Toenail Se, 16 weeks (μg/g)∥	217	0·34	0·164, 0·72	0·005	217	0·38	0·17, 0·87	0·021	210	0·32	0·13, 0·78	0·013
Treatment (Se *v.* placebo)	227	0·39	0·15, 1·06	0·066	217	0·35	0·11, 1·08	0·067	210	0·30	0·09, 1·00	0·049
Age (years)	227	0·99	0·88, 1·10	0·818		NA§			NA§	
History of PE in mother	227	0·67	0·08, 5·38	0·710		NA§			NA§	
History of PE in sister	227	1·76	0·20, 15·42	0·608		NA§			NA§	
Whole-blood Se, 12 weeks (μmol/l)	227	0·81	0·13, 5·00	0·816		NA§			NA§	
Whole-blood Se, 35 weeks (μmol/l)	215	0·39	0·13, 1·22	0·106		NA§			NA§	
SEPP1, 35 weeks (mg/l)	215	0·72	0·51, 1·01	0·060		NA§			NA§	

BP, blood pressure; NA, not applicable; SEPP1, selenoprotein P.

* Risk factors for the development of PE/PIH assessed individually by logistic regression.

† Optimal model of risk factors for the development of PE/PIH assessed by forward logistic regression, including diastolic BP, BMI, toenail Se concentration and treatment.

‡ Optimal model of risk factors for the development of PE/PIH as in model 1, but excluding those Se-treated women who took < 60 % of their treatment pills.

§ Variable was not selected in the stepwise analysis.

∥ Variable multiplied by 10 for scaling purposes.

Based on adjusted model 2 (Table 3), women with 1 mm Hg higher DBP at baseline were 1·12 times more likely to develop PE/PIH than other women, those with 0·1 μg/g higher toenail Se concentration were only 0·32 times as likely to develop PE/PIH than other women, and those who consumed 60 % or more of their Se tablets were only 0·30 times as likely to develop PE/PIH than women who took placebo.

The median toenail Se concentration in women who developed PE/PIH was significantly lower than that in other women (0·57, range 0·46–0·64 *v.* 0·61, range 0·46–1·11; *P*= 0·004).

## Discussion

### Baseline selenium status

We chose to measure whole-blood Se concentration rather than serum/plasma Se concentration, as it is a longer-term measure of Se status and therefore less subject to day-to-day variation. However, this means that we have far fewer pregnancy data with which to compare our values. There is only one reported first-trimester value of approximately (read from a graph) 1 μmol/l^(^ ^22^ ^)^, which is lower than our baseline (12-week) value of 1·31 μmol/l, but it was reported from a study in Serbia, a known low-Se area^(^ ^23^ ^)^. Other reported values are from the third trimester until delivery, and range from 0·76 to 0·8 in Serbia^(^ ^22^ ^,^ ^24^ ^)^, 1·30 to 1·36 in Kuwait^(^ ^25^ ^,^ ^26^ ^)^, to 1·51 μmol/l in Da-Ye, China^(^ ^27^ ^)^, and can be compared with the median value of 1·16 μmol/l found in the present study for the placebo group at 35 weeks of gestation. Hence, whole-blood Se concentration in our pregnant population was a little lower than the middle of the range observed in the few other populations for whom data were available.

The median toenail Se concentration in clippings taken at 16 weeks of gestation (laid down before conception) was 0·61 μg/g, almost identical to the median value of 0·62 μg/g found in pregnant controls in our previous study^(^ ^17^ ^)^. This value is similar to that of toenail Se concentrations measured in women from The Netherlands (mean 0·58–0·72 μg/g), but considerably lower than in women from the USA (mean 0·75–0·92 μg/g)^(^ ^17^ ^)^.

The median baseline GPx3 activity was 72·4 units/l (Table 1). This is considerably lower than other values of GPx3 activity measured in other studies of pregnant women in the same laboratory, i.e. mean GPx3 activities of 84 and 104 units/l were found in cohorts of US and Australian women in the second trimester of pregnancy, respectively (Vanderlelie *et al.*, unpublished results). These values can more properly be compared with the value of 75·4 units/l reported in the present study for the placebo group in the second trimester. Hence, it can be concluded that GPx3 activity was low in this UK pregnant population, probably reflecting a Se intake that is insufficient to optimise plasma GPx activity^(^ ^28^ ^,^ ^29^ ^)^.

Women who were older, of higher social class (based on occupation) and left education at a later age had significantly higher Se status (whole blood and toenails). These findings are in agreement with those of the 2001 Adult UK National Diet and Nutrition Survey where higher plasma Se concentration was associated with older age, better education and higher earnings (*P*≤ 0·001)^(^ ^30^ ^)^.

With regard to diet, the consumption of Brazil nuts, known to be high in Se, significantly increased whole-blood Se concentration, although its significant effect on toenail Se concentration disappeared after adjustment; however, the number of Brazil nut consumers was small (*n* 26). The consumption of seafood tended to increase both whole-blood and toenail Se concentrations.

### Effect of treatment: longitudinal effects on status

As expected, there was a significant increase in whole-blood Se concentration in the Se-treated group from 12 to 35 weeks of gestation. This reflects both an increase in the concentrations of the plasma selenoproteins, SEPP1 and, to a lesser extent, GPx3 (see below) and, to an unknown degree, a non-specific incorporation of Se as selenomethionine into proteins including albumin and erythrocyte Hb. Over the corresponding period, a significant decrease in whole-blood Se concentration was observed in the placebo group, which can be interpreted as partly due to the increase in plasma volume that occurs in pregnancy and partly due to placental transfer of Se to the fetus by SEPP1 via the apoER2 receptor^(^ ^9^ ^)^. It would appear that pregnancy is putting pressure on the Se stores of these women whose Se status may be inadequate to deal with fetal demands.

Women in the Se-treated group had a median SEPP1 concentration that was 77 % higher at 35 weeks than those in the placebo group. Baseline SEPP1 concentration was quite low in this cohort, and even after supplementation, the suggested plateau at 6·5–7 mg/l was not reached in many of the supplemented women, suggesting a requirement for a higher dose or longer treatment period^(^ ^9^ ^)^.

In Se-supplemented women alone, a small but significant increase in GPx3 activity was found between 12 and 20 weeks, which was also observed between 12 and 35 weeks, despite a modest decrease between 20 and 35 weeks. Although this might imply that GPx3 activity was already almost maximised at baseline, this interpretation does not fit with the fact that GPx3 activity in UK pregnant women was considerably lower than that in other cohorts measured in the same laboratory. It is more likely that additional Se is being prioritised for the synthesis of other selenoproteins, notably SEPP1, which at 35 weeks was substantially higher in the Se-treated group than in the placebo group. Studies in both cell and animal models have suggested that a hierarchical selenoprotein expression pattern occurs during deprivation and supplementation states^(^ ^31^ ^–^ ^33^ ^)^. Furthermore, as mitochondrial oxidative stress increases over the course of pregnancy, intracellular GPx isoforms may be preferentially expressed over extracellular GPx3 for the protection of the trophoblast^(^ ^34^ ^,^ ^35^ ^)^.

### Risk of development of hypertensive conditions of pregnancy

We combined the outcomes of PE and PIH because the distinction between the two is not always completely clear; 15–45 % of women with PIH will eventually develop PE^(^ ^36^ ^,^ ^37^ ^)^, particularly when PIH presents early in gestation^(^ ^38^ ^)^. Indeed, PIH associated with features of PE other than proteinuria, such as hyperuricaemia or intra-uterine growth retardation, is now regarded as an atypical variant of PE^(^ ^39^ ^)^. Furthermore, there are immunological similarities between the conditions, both of which show a general depression in immunocompetent lymphocytes with a higher overall level of T-cell activation^(^ ^40^ ^)^, suggesting some commonality in aetiology. Increased oxidative stress is also a feature observed in both conditions^(^ ^41^ ^,^ ^42^ ^)^.

We previously found that women supplemented with Se had a significantly lower risk of the combined hypertensive outcomes than those treated with placebo (adjusted OR 0·35, *P*= 0·044)^(^ ^9^ ^)^. PE is associated with increased systemic (vascular) inflammation relative to normal pregnancy^(^ ^43^ ^)^; although this has not been shown for PIH as such, the effect of Se on the risk of developing PE/PIH may be partly due to the ability of selenoproteins to counteract inflammation^(^ ^44^ ^,^ ^45^ ^)^. Se supply is known to affect the synthesis and actions of eicosanoids, important modulators of inflammation, platelet activation, blood pressure and the immune response^(^ ^44^ ^,^ ^45^ ^)^; for instance, under oxidative stress, Se-deficient endothelial cells produce less of the vasodilatory eicosanoid, prostacyclin^(^ ^45^ ^)^.

A further important anti-inflammatory mechanism of Se involves selenoprotein S (SELS). SELS manages inflammation control in the endoplasmic reticulum by assisting in the retrotranslocation of misfolded proteins from the endoplasmic reticulum into the cytosol^(^ ^46^ ^)^. Endoplasmic-reticulum stress is known to be involved in the pathophysiology of PE^(^ ^47^ ^)^. A SNP in the gene encoding SELS has been correlated with serum concentrations of pro-inflammatory cytokines^(^ ^48^ ^)^ and with the risk of developing PE in a large Norwegian cohort^(^ ^16^ ^)^.

Of the known risk factors for hypertensive conditions of pregnancy^(^ ^7^ ^)^ treated individually, only baseline DBP and BMI reached significance in the present study cohort. Of the Se-related risk factors, toenail Se, which measures pre-pregnancy Se status, was by far the most significant determinant of PE/PIH. When only significant or near-significant factors were entered into a stepwise model, toenail Se became a more significant determinant with a larger effect size than BMI; after exclusion of Se-treated non-compliers, Se treatment also significantly reduced the odds of developing PE/PIH.

The strong effect of pre-pregnancy Se status is reflected in the significant difference in median toenail Se concentration between women who developed PE/PIH and other women. This result is consistent with our previous finding in the same region of the UK demonstrating significantly lower toenail Se concentration in women with PE than that in matched pregnant controls^(^ ^17^ ^)^.

What can we conclude from these findings and particularly from the fact that pre-pregnant Se status (as measured in toenails) appears to be a more significant predictor than Se treatment started at 12 weeks? If Se is having its effect through antioxidant selenoproteins – it is known, for instance, that it has the capacity to up-regulate antioxidant systems and protect trophoblast cells from oxidative stress^(^ ^49^ ^)^ – it would need to be present at the time of a relevant pro-oxidant challenge^(^ ^50^ ^)^. Such a pro-oxidant challenge occurs at the initiation of intervillous blood flow at 8–10 weeks of gestation that is associated with a burst of oxidative stress^(^ ^42^ ^,^ ^51^ ^)^. Se supplementation did not start until 12 weeks and then only at a low dose, so it would have taken some time for antioxidant selenoenzymes to reach an adequate protective level; non-pregnant individuals supplemented with 100 μg Se, as selenomethionine, took 60 d to reach a plateau in GPx3 activity^(^ ^52^ ^)^.

Alternatively, is pre-pregnancy Se status more important in preventing hypertensive pregnancy conditions than status in pregnancy itself? The development of the oocyte, fertilisation and implantation are periconceptual events that could be affected by Se status^(^ ^53^ ^,^ ^54^ ^)^. Antioxidant selenoproteins can counteract excessive synthesis of reactive oxygen species that impairs oocyte maturation and embryo development^(^ ^53^ ^,^ ^55^ ^)^. The fact that periconceptual multivitamin/mineral use reduces the risk of developing PE suggests that an adequate status of protective vitamins and minerals is important before, or shortly after, conception^(^ ^56^ ^,^ ^57^ ^)^.

Se status in very early pregnancy might also affect PE/PIH through an effect on regulatory T (T_reg_) cells that are necessary, at least in the mouse, to maintain materno-fetal tolerance and a normal pregnancy outcome^(^ ^58^ ^,^ ^59^ ^)^. Indeed, T_reg_ cells are essential even before embryo implantation^(^ ^60^ ^)^. Dietary Se has been shown to increase the differentiation of naive CD4^+^ T cells into CD25^+^Foxp3^+^ T_reg_ cells in mice^(^ ^44^ ^)^, while Se treatment significantly increased the percentage of T_reg_ cells and the expression of *Foxp3* mRNA in a mouse model of autoimmune thyroid disease^(^ ^61^ ^)^.

A limitation of the present study was that it was not designed to determine hypertensive outcomes of pregnancy, therefore the study was not adequately powered for that end point. Although we attempted to capture the intake of Se-rich foods through a FFQ, the results need to be interpreted with caution because of the inherent limitations of the FFQ.

### Conclusion

We validated our first hypothesis that UK pregnant women have low Se status, as determined by the median value of a number of status parameters. We also validated our second hypothesis that Se status/Se supplementation affects the risk of developing hypertensive conditions of pregnancy; the odds of PE/PIH were significantly reduced in women with higher pre-pregnancy Se status (toenail Se) and in those supplemented with Se from 12 weeks of gestation.

In conclusion, UK women need to improve their Se status, ideally before pregnancy, as our data suggest that pre-pregnancy status has a significant effect on the risk of developing hypertensive conditions of pregnancy. Although our simplified FFQ revealed that consumption of Brazil nuts was associated with higher whole-blood Se concentration, we cannot recommend them as a means of increasing Se status as they contain both Ba, at a level where consumption of two nuts per d could exceed the reference dose, and Ra (Ra^228^), with an activity of 6–133 Bq/kg^(^ ^62^ ^–^ ^64^ ^)^. Offal and seafood are other rich sources of Se^(^ ^65^ ^)^ and higher consumption of seafood was marginally associated with better Se status; however, these foods are often unpopular. Hence, it is likely that many women will need to take a Se-containing supplement (e.g. as part of a multivitamin and mineral pregnancy supplement) at least as soon as they know they are pregnant, and preferably when planning pregnancy. We gave a supplement of 60 μg Se/d; however, a dose of 100 μg Se/d would probably be preferable in women who only commence supplementation when pregnancy is confirmed, as even at that higher dose, 60 d were required for a plateau to be reached in GPx3 activity^(^ ^52^ ^)^.

A study in a larger cohort of pregnant women is needed to confirm these findings.
